# Development of Composite Thermocouple Materials Using PEDOT:PSS and Bi_2_Te_3_ for Wearables Thermopiles

**DOI:** 10.3390/ma18215046

**Published:** 2025-11-05

**Authors:** Olga Rac-Rumijowska, Piotr Markowski, Karol Rauch, Patrycja Suchorska-Woźniak, Andrzej Dziedzic

**Affiliations:** 1 Faculty of Electronics, Photonics and Microsystems, Wrocław University of Science and Technology, Wybrzeże Wyspiańskiego 27, 50-370 Wrocław, Poland; piotr.markowski@pwr.edu.pl (P.M.); patrycja.suchorska-wozniak@pwr.edu.pl (P.S.-W.); andrzej.dziedzic@pwr.edu.pl (A.D.); 2 Faculty of Fundamental Problems of Technology, Wrocław University of Science and Technology, Wybrzeże Wyspiańskiego 27, 50-370 Wrocław, Poland; karol.rauch@pwr.edu.pl

**Keywords:** thermocouple, screen-printing, wearable electronics, PEDOT:PSS, bismuth telluride (Bi_2_Te_3_), wearable thermopile, seebeck effect

## Abstract

This paper presents results on the preparation of thermoelectric composite materials for flexible and wearable electronics applications. Composite materials in the form of pastes for screen printing or stencil printing were made from a mixture of PEDOT:PSS paste and Bi_2_Te_3_ powder. The pastes showed good adhesion both to polyimide foil (Kapton) and polyester fabric substrates. Depending on the composition and the substrate used, the pastes had a sheet resistance of 26–264 Ω/sq, a Seebeck coefficient of 14–45 μV/K and a power factor of 0.05–0.8 μW/mK^2^. The obtained pastes enabled the fabrication of textile thermopiles using Ag and PEDOT:PSS/Bi_2_Te_3_ materials for both arms. The output voltage of the obtained thermopiles on textile and foil substrates was 6–8 mV at a temperature gradient of 100 °C, and the output power was 0.01–0.12 μW. Energy harvesting from the human–ambient temperature gradient using the developed generators yielded promising results, with output voltages around 0.3 mV.

## 1. Introduction

Recently, there has been a huge development in wearable electronics, which is based on producing various types of electronic components in a form that can be worn on the body or attached to clothing. These components have applications in medicine, sports, military or everyday life. Their purpose is the non-invasive and continuous monitoring of such parameters as body temperature, sleep apnoea or stress levels [1,2,3] in, for example, the elderly, the sick or infants. In the context of an ageing population and a shortage of care staff, the importance of wearable sensors for monitoring vital signs should be highlighted [4].

However, despite the huge interest in wearable electronics, which also has an economic translation—a market value of USD 120.54 billion in 2023 [5]—and application potential in various areas of life (sports, health, military), there are still many unsolved scientific and technological problems. The majority of commercially available wearables are still based on external accessories such as watches, wristbands and bracelets, rather than on the production of textile electronic components. This is because the creation of stable, flexible, wearable and comfortable electrical connections is still an unsolved problem. In addition, there is a problem with standard electronics technology—the power supply, which, despite the miniaturization of devices and their power consumption, requires the installation of batteries or rechargeable batteries whose weight and size far exceed that of the device itself. On the other hand, it is extremely important to focus attention on the development of novel ideas for wearable power supply and energy harvesting [6,7], which would allow wearable devices to be powered by energy harvested from the environment.

One of the physical effects that make it possible to power electrical circuits with extracted energy are thermocouples and thermopiles based on the Seebeck effect. Thermopiles are assemblies of thermocouples which are electrically connected in series and thermally connected in parallel. This means that the thermoelectric force generated by the single thermocouple is multiplied by the number of junctions used. Thermocouples are widely and commercially used both as highly sensitive (20–50 µV/K) temperature sensors [8] and as component of power generators for space probes and other applications [9,10]. Due to the proliferation of portable electronics, there is great interest in the manufacture of portable thermocouples [1].

A thermocouple is an element of an electric circuit consisting of two different materials (mainly conductors and/or semiconductors with different values of the Seebeck coefficient—α_A_, α_B_ [V/K]) and using the Seebeck effect occurring at their contact. Due to the temperature difference between the junctions (measuring and reference, so-called cold and hot ends of the thermocouple—T_1_, T_2_ [K]), a potential difference (electromotive force) is created, called thermoelectric force—E_T_ [V], proportional to the temperature difference (1). Thermocouples are connected electrically in series (n—number of thermocouples) in thermopiles to increase the voltage generated E_OUT_ [V] (2).

(1) $$E_{T}=\left(\alpha_{A}-\alpha_{B}\right)\left(T_{1}-T_{2}\right)$$

(2) $$E_{OUT}={n\;\cdot \;E}_{T}$$

The suitability of materials for thermoelectric components, especially voltage generators, is assessed on the basis of the thermoelectric power factor PF [W∙m^−1^∙K^−2^] (3).

(3) $$PF=\alpha^{2}\cdot \sigma$$

It implies that materials must have both a high electrical conductivity (σ) [S∙m^−1^] and a high Seebeck coefficient (α). These values are dependent on each other, as both depend on the amount and mobility of the carriers. For this reason, an increase in one always results in a decrease in the other. These parameters are also correlated with the thermal conductivity of the material λ, which is the sum of the electron (λe) and phonon (λf) components. In order to reduce λ, dopants that disrupt (defect) the crystal lattice are introduced into the material. As a result, the phonon component decreases without changing the electron component. This is crucial because a change in the electron component would equally affect the electrical conductivity. On the other hand, phonon interactions are one of the factors determining the magnitude of the Seebeck coefficient.

A more accurate parameter for determining a material’s ability to generate a thermoelectric voltage is the Z-value (figure of merit) [K^−1^] (4), which combines the three most important parameters characterizing thermoelectric materials—Seebeck coefficient α, electrical conductivity σ and thermal conductivity λ [11].

(4) $$Z=\frac{\alpha^{2}\cdot \sigma }{\lambda }\;$$

For temperature sensors, the most important the Seebeck coefficient of both materials used for thermocouple arms (and their difference), while for thermoelectric generators it is important to determine the thermoelectric power factor PF and dimensionless figure-of-merit Z values, from which it follows that materials must have both high electrical conductivity (σ) [S∙m^−1^], high Seebeck coefficient (α) and low thermal conductivity (λ) [W∙m^−1^∙Km^−1^] to be good thermoelectric materials. Due to the low conductivity, when heating one end of the thermocouple, the other end remains cold. This helps generate a larger temperature gradient and thus a greater thermoelectric force.

Research into the fabrication of thermopiles in terms of sensing properties and voltage generation focuses on modifying the materials for the thermocouple arms are made [12,13,14,15,16,17] (type of material, size, dopant) or modifying their structure (thermopiles, 3D structures, geometry) [18]. In contrast, the majority of thermopiles are still made on rigid substrates (ceramic, silicon). Relatively few reports deal with the fabrication of thermocouples on flexible substrates, in particular on textile substrates. Most textile thermocouples reported in the literature are based on the weaving of metal wires and fibres into the structure of textiles, which reduces their flexibility [19,20] and causes discomfort in use [21]. Many textile thermocouples are characterized by significant drift and fluctuation of the generated voltage [22].

Most publications in the field of textile thermocouples present preliminary results concerning the characterization of the thermoelectric materials themselves on fabrics, the preparation of thermocouples and possibly their application as temperature sensors [23]. But thermocouples and thermopiles, on the other hand, have the potential to be used in textiles not only as temperature sensors but also as thermoelectric generators. When incorporated into clothing, they can be used to provide the voltage and power required by portable electronic devices. This is possible because there are areas on the human body with a natural temperature gradient caused by the use of electronics [24]. Moreover, it is possible to exploit the temperature difference between the human body and the clothing. By using suitable thermoelectric materials, it is possible to obtain thermocouples with an output power that can be used in energy generators on foil substrates. Zhuo Cao et al. [25] investigated the possibility of fabricating thermocouples from tellurium-antimony (Sb_2_Te_3_) combined with (Bi_1.8_Te_3.2_) via silver paste or undoped tellurium-antimony, all samples fabricated on flexible Kapton foil. The resulting thermocouples gave a signal of a few mV with a temperature change of about 10 °C [26] and about six times the response of n-type doped Bi_2_Te_3_ combined with p-type Sb_2_Te_3_ [27]. In contrast, analogous literature reports on textile substrates are still lacking.

One technique that makes it possible to create conductive elements on textiles that do not require the incorporation of metal wires and yarns is screen or stencil printing. Screen printing is a method known and used for fabric modification already in the Song Dynasty in China (960–1279 AD). For many years it was used only to give colours or prints to fabrics. However, at the beginning of the 20th century it began to be used in a completely new area—electronics, for the production of thick layers. With the development of printed electronics it was possible to obtain thick layers not only on thermally resistant ceramics substrates, but also thanks to the use of polymer pastes on flexible substrates. In recent years the screen printing method of conductive layers on textile substrates is investigated and applied to wearable electronics [28,29]. However, this requires the development of new composite materials (pastes) that allow for the effective application of prints and at the same time have sufficient electrical (or thermoelectric) properties. Composite materials, such as screen printing pastes, consist of two phases—the functional phase (the material that imparts the final electrical properties in the form of nano- and/or micro-powders) and the carrier (the polymeric material that binds the particles of the functional phase together, allowing printing and adhesion to the substrate). For all thermoelectric parameters, it is important to consider the role and influence of both components of the paste, as bulk materials have different properties to composites (pastes) [30]. In conductive composite materials, even when the percolation threshold is exceeded, there is always the phenomenon of current tunnelling through the thin polymer layer surrounding the conductive particles. For this reason, even when the percolation threshold is exceeded, the electrical conductivity of the composite is always 2–4 orders of magnitude lower than the conductivity of the functional material used [31,32], and analogous behaviour can be observed for the other parameters relevant to thermoelectric properties—Seebeck coefficient and thermal conductivity.

The literature provides examples of thermoelectric materials for wearable electronics applications. Lu et al. fabricated composite films made of PEDOT:PSS on a nylon membrane [33], Song et al. layered nanostructure PEDOT:PSS/SWCNTs [34], Liu et al. PEDOT:PSS/silicon dioxide nanoparticles composite films [35] and Rathi et al. PEDOT:PSS/Bi_2_Te_3_/reduced graphene oxide ternary composite films [36]. The article presents the properties of the thermoelectric screen-printed films based on bismuth telluride (functional phase), while the conductive polymer PEDOT:PSS, which is also characterized by good thermoelectric properties, was used as the carrier phase. The pastes were applied to textile (polyester—PES) and polyimide foil (Kapton) substrates. In order to make a second thermocouple arm, analogous prints were made from a commercial silver-based conductive paste. The obtained pastes enabled the fabrication of textile thermopiles using Ag/PEDOT:PSS/Bi_2_Te_3_ materials.

## 2. Materials and Methods

### 2.1. Raw Materials and Paste Preparation

The silver thermocouple arm is made of conductive DuPont PE874 screen printing paste from the Intexar series intended for flexible substrates, including textile.

To prepare the paste for the second arm of the thermocouple 325 mesh powder of bismuth telluride Bi_2_Te_3_ 99.99% trace metal base (Sigma Aldrich, St. Louis, MO, USA) and Poly(3,4-ethylenedioxythiophene)-poly(styrene sulfonate) (PEDOT:PSS) 5.0 wt.%, conductive screen printable ink (Sigma Aldrich) was used.

**Figure 1:**
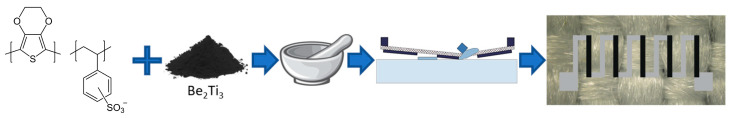
Schematic diagram of obtaining materials.

Pastes containing a mixture of bismuth telluride powder and PEDOT:PSS paste were prepared by mixing them in a mortar until a homogeneous consistency was obtained (approximately 5 min). The weight ratio of the components in the individual samples is presented in Table 1. The scheme for obtaining the materials is shown in Figure 1.

Polyimide foil DuPont™ (Wilmington, DE, USA) Kapton (thickness 125 μm) and polyester fabric (thickness 150 μm) no. 205509/AN/BS PES 100% from Miranda Limited liability company (Turek, Poland) were used as a substrates.

### 2.2. Printing Procedure

**Figure 2:**
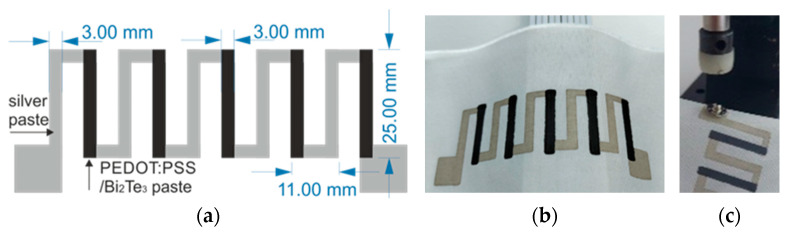
Schematic diagram (**a**), photography (**b**) and electrical connection (**c**) of the thermopile (grey colour—silver paste; black colour—PEDOT:PSS/Bi_2_Te_3_ paste).

All prints were made on polyimide foil and polyester fabric substrates with dimensions of 100 × 150 mm^2^. First, test samples were printed from each paste—5 rectangular strips of 28 × 3.5 mm^2^ (8 squares), then thermopiles consisting of five thermocouples were made from one of obtained pastes (Figure 2), in which the electrical contacts and one of the thermocouple arms were made from silver paste and the other from PEDOT:PSS/Bi_2_Te_3_ paste. The films from PE874 paste were screen-printed with the aid of semi-automatic screen printer Uniprint Go3V (PBT WORKS, Rožnov pod Radhoštěm, Czech Republic). A 200 mesh stainless screen was used to make the prints, on which a photosensitive emulsion (thickness 30 μm) with a pattern was applied. PEDOT:PSS/Bi_2_Te_3_ pastes were applied using the stencil printing method. The stencil was made of 0.25 mm thickness PET foil known under the trade name Melinex (Selmex company, Plewiska, Poland). Based on our previous studies [37], four prints on fabric substrates and two prints on foil were made, in order to optimally cover the surface. After printing, all layers were cured for 15 min at 130 °C at a well-ventilated dryer Binder FED-56 (Binder Gmbh, Tuttlingen, Germany) according to data sheet of used materials. The electrical connections to the fabric were made using 10.5 mm snaps (Figure 2c). The lower part of the snap was attached to the fabric using a leatherwork press, and the interior of the snap was filled with PE874 paste. After crimping, the structure was dried (15 min, 130 °C). The upper part of the snap was connected to the wire by soldering.

### 2.3. Characterization

**The morphology** of Bi_2_Te_3_ powder by Scanning Electron Microscope (SEM) (Hitachi SU6600, Tokyo, Japan) when as the morphology of printed and cured films was investigated using Leica DM4500 B LED optical microscope (Wetzlar, Germany) and morphology. The film thickness of the prints was determined using a Keyence VHX 7000 digital microscope (Keyence International, Mechelen, Belgium), which allows 3D measurements of the objects and the calculation of its height profiles, as well as electronic micrometer gauge.

**Electrical conductivity** was measured using the Ossila Four-Point Probe System (Sheffield, UK) with Ossila Sheet Resistance software Ossila Sheet Resistance v. 2.1.1. The bench uses four probes lying in a line at equal intervals and made of the same metal alloy. The contact points face the substrate giving the possibility of current flow through the blades. The inner electrodes are designed to measure the potential difference arising in the material. As a result of the current *I* flowing through the film, a potential distribution *U* is formed in the film, which is strictly dependent on the resistivity of the material and the geometry of the sample. It is therefore important to keep the measurement location and the geometrical dimensions of the sample constant. The direct measurand is the surface resistance *R* expressed by Formula (5), while the resistivity of a film of thickness *d* is determined by Formula (6):

(5) $$R=\frac{U}{I}[\Omega ],$$

(6) $$\varrho =K\;\frac{U}{I}d\;[\Omega \cdot m],$$

where *K* is the correction factor. It is related to the geometry of the substrate and the dimensions of the measuring head and is calculated theoretically. In the case of the present measurements, *K* = 0.61.

**Temperature coefficient of Resistance (TCR)** was calculated using Formula (7). Resistance measurements were carried out using Keysight Technologies 34461A multimeter (Keysight Technologies, Santa Rosa, California, United States) by technical method. The samples were heated to 125 °C on a heating plate of our own manufacture, in increments of 5 °C.

(7) $$HTCR=\frac{R\left(125\;{}^{\circ}C\right)-R\left(25\;{}^{\circ}C\right)\times 10^{6}}{R\left(25\;{}^{\circ}C\right)\times 100}\;[ppm/K],$$

where *R* (125 °C)—resistance at 125 °C; *R* (25 °C)—resistance at 25 °C; and HTCR—Hot Temperature Coefficient of Resistance.

**Figure 3:**
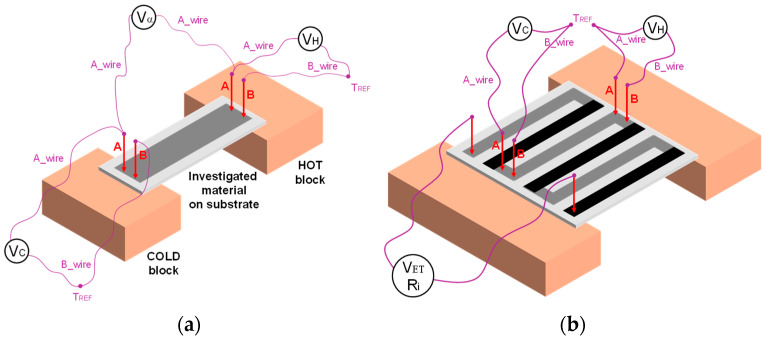
Schematics of the set-ups used to measure: (**a**) the Seebeck coefficient of the materials; (**b**) the output parameters of the thermopiles.

**The determination of the Seebeck coefficient** of the tested materials was carried out using an automated set-up (Figure 3a) [38,39]. It consists of two copper blocks acting as a heater (HOT) and a heat sink (COLD), three voltmeters, probes and measuring wires made of a reference thermoelectric materials. The heater temperature is adjustable from room temperature to 220 °C. The heat sink temperature is stabilized to near-room temperature. The substrate with the test sample is placed on copper blocks (Figure 2a). Two measuring probes (A and B) made of a reference thermoelectric materials are attached to the hot part of the test material. These were NiCr (probe A) and NiAl (probe B). Together they form a K-type thermocouple. To probes A and B measuring wires are connected (A_wire and B_wire), made of the same materials. The A_wire and B_wire are short-circuited at a location with a known reference temperature, *T_REF_*, forming the cold junction of the measuring thermocouple. The hot junction is formed by the electrical short-circuit of probes A and B through the material investigated. If the contact temperatures of junctions probe A/sample and probe B/sample are the same (it is met when the contacts are close together), then the temperature at the junction probe A/sample (*T_HOT_*) can be determined very accurately using the *V_HOT_* voltmeter. Similarly, the temperature of the junction probe A/sample on the COLD block (T_COLD_) is determined. A third voltmeter (*V_α_*) measures the electromotive force (*E_T_α_*) generated by a thermocouple consisting of the investigated material and the thermoelectric material A (NiCr), with a known Seebeck coefficient (α_A_). Hence, the Seebeck coefficient of the investigated sample (α_sample_) can be determined using Formula (8):

(8) $$E_{T\_\alpha }=\frac{\left(\alpha_{sample}-{\;\alpha }_{A}\right)}{\left(T_{HOT}-T_{COLD}\right)}$$

The determination of the output parameters of thermopiles composed of five thermocouples was performed on a similar set-up (Figure 3b). To accurately determine the temperatures of the cold and hot sides of the thermopiles, probes and measuring wires made of thermoelectric reference materials A and B (NiCr and NiAl) were used. They were mounted on the arms of the thermopiles, analogous to the measurements described above. In addition, a multimeter measuring the generated *V_ET_* voltage and the internal resistance *R*_i_ was attached to the contact fields of the thermopile. The heater was heated from room temperature to approximately 125 °C, allowing the characteristics *V_ET_* = f(*T_HOT_*), *R_i_* = f(*T_HOT_*) and *P_OUT_* = f(*T_HOT_*) to be determined.

## 3. Results and Discussion

### 3.1. Morphology of Materials

**Figure 4:**
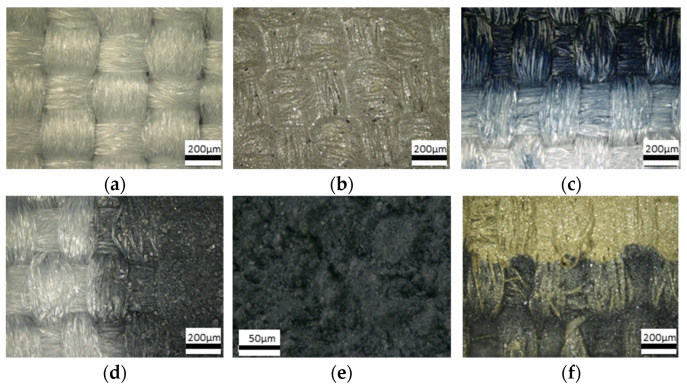

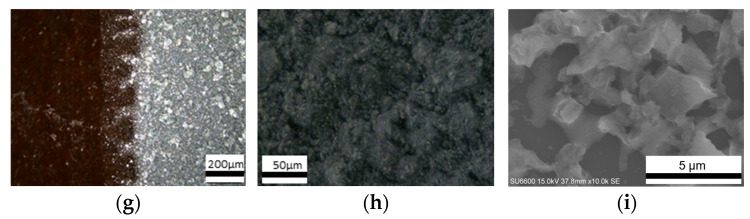
Microstructure of the (**a**) clear surface of textiles; textiles covered by (**b**) silver paste, (**c**) PEDOT:PSS paste, (**d**,**e**) paste containing 70:30 PEDOT:PSS:Bi_2_Te_3_; (**f**) border of print from paste PEDOT:PSS and 70:30 PEDOT:PSS:Bi_2_Te_3_; (**g**,**h**) polyimide foil covered by paste 70:30; (**i**) SEM image of Bi_2_Te_3_ powder.

The microstructure of the resulting films was determined by optical microscopy. As a result of printing the paste four times on textile substrates and two times on film substrates, full surface coverage was obtained, both for fabric and foil (Figure 4b–h). However, in the case of printing on fabric, the paste penetrates almost the entire depth of the fabric. The printed surface on the fabric accurately reproduced the structure of the pure fabric (Figure 4a) for all silver paste (Figure 4b), PEDOT:PSS paste (Figure 4c) and 70:30 PEDOT:PSS/Bi_2_Te_3_ paste (Figure 4d,e). A photograph was also taken of an example of the bonding area between the two pastes (silver and 70:30 PEDOT:PSS/Bi_2_Te_3_) (Figure 4f) to show the good contact between the pastes applied to the textile substrate.

SEM image (Figure 4i) of Bi_2_Te_3_ powder shows that materials is a powder form with a grain size of 5–50 μm, these particles are visible in pastes containing bismuth telluride (Figure 4d,e,g,h) regardless of the type of substrate, in contrast to the polymeric PEDOT:PSS paste (Figure 4c) forming a uniform, continuous and thin film.

### 3.2. Film Thickness

**Figure 5:**
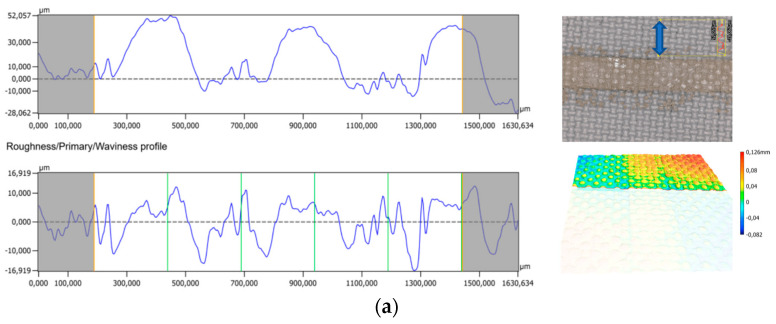

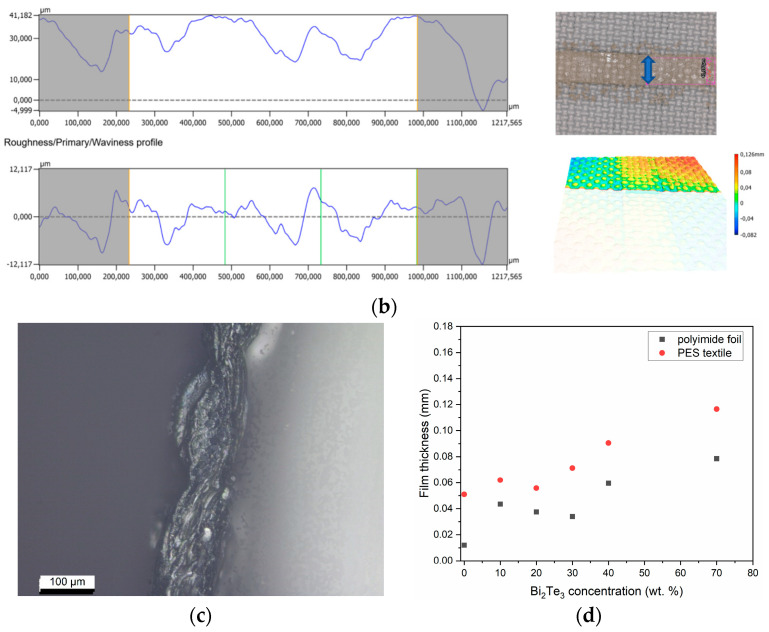
Profilometry analysis of (**a**) uncovered textile; (**b**) textile with printed silver film; (**c**) optical microscope image of a cross-section of a silver print on fabric; and (**d**) average dependence of print thickness on paste composition.

Determining the thickness of prints in typical thick films is relatively simple. However, this is not the case for prints made on fabrics. Due to the required viscosity of the screen-printing paste to enable it to be applied to the substrate, the paste largely penetrates the highly absorbent textile substrate. On the one hand, this phenomenon makes it possible to obtain films with high adhesion to the substrate; on the other hand, it makes it significantly more difficult to determine the thickness of the films, which is essential for determining the electrical parameters of the material. The profilometric measurements taken of the clean (Figure 5a) and paste-coated fabric (Figure 5b) did not allow a clear measurement of the film thickness but only showed that the fabric was smoother after printing. This is related to the complete absorption of the paste into the fabric, which is visible on the cross-section of the print (Figure 5c). For this reason, it is not possible to separate the thickness of the print from the thickness of the substrate.

However, determining the resistivity of the material is essential to determine the quality of the thermoelectric material. For this reason, it was decided to take approximate measurements of the printed thicknesses, which consisted of measuring the printed films five times using an electronic micrometre screw. The thickness of the substrate was then subtracted from the averaged value, respectively, 150 μm for the PES fabric and 125 μm for the polyimide film. The dependence of the average film thickness on the sample composition (Figure 4d) shows that the films thickness increases slightly with the bismuth telluride content. This is related to the increasing density and viscosity of the PEDOT:PSS paste when Bi_2_Te_3_ powder is added to it. The average thickness of the layers obtained on the film was 44 μm and, on the fabric, 75 μm, which is related to the application of two prints of paste on the foil and four prints on the fabric.

### 3.3. Electrical Conductivity

**Figure 6:**
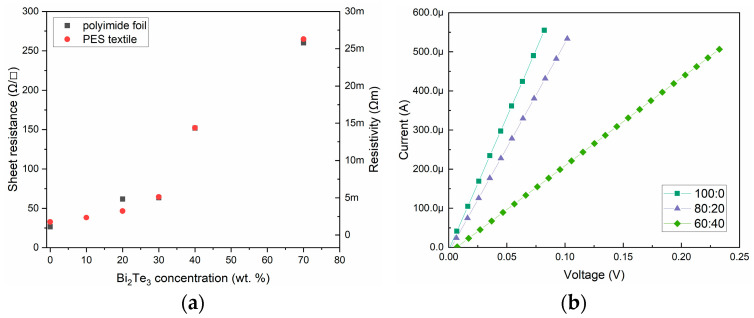
(**a**) Change in paste conductivity as a function of bismuth telluride concentration. (**b**) Current voltage characteristics of the obtained pastes.

The measurements of the sheet resistance showed that the content of conductive polymer in the paste has a dominant effect on the conductivity of the material. The addition of increasing amounts of semiconducting Bi_2_Te_3_ significantly increases the resistance of the paste (Table 2) (Figure 6a). Furthermore, all the pastes, irrespective of the type of substrate they were applied to, exhibited linear current-voltage characteristics, which is indicative of the electronegative nature of the paste conductivity (Figure 6b). Silver-based PE 874 film has a sheet resistance of 0.22 Ω/sq on PES textiles substrate and 0.12 Ω/sq on polyimide foil. The resistivity is higher than declared by the manufacturer, which is due to the different thickness of the actual layers made.

### 3.4. Temperature Coefficient of Resistance

**Figure 7:**
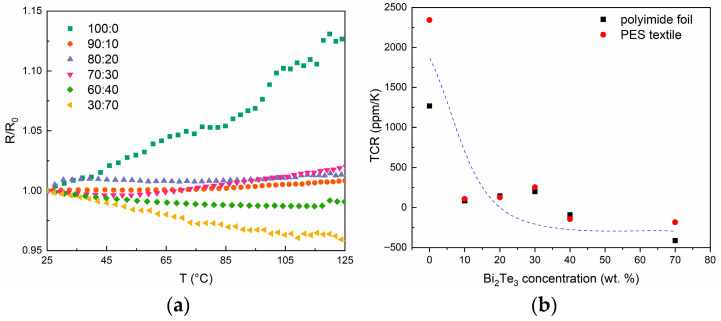
(**a**) Changes in resistance (R/R_0_) during heating of samples printed on polyimide foil, (**b**) plot of TCR versus bismuth telluride concentration for samples made on foil and fabric.

The temperature coefficient of resistance (TCR) is a parameter that characterizes metals and semiconductors. It describes how the resistance of a material changes when its temperature is increased by 1 K. The TCR for a paste containing only conductive PEDOT:PSS has the highest value, which gradually decreases with increasing bismuth telluride content. For a sample containing as much as 40 wt.% Bi_2_Te_3_, the TCR value decreases to negative values, indicating a dominance of conductivity by the semiconductor. This trend is evident for both substrates (Table 3, Figure 7).

However, differences in TCR are apparent for the same paste, but applied as film on different substrates. These differences are due to the different film structure on the different substrates. On polyimide foil, the layer is applied to the surface, while in the case of fabric, it is the measurement of the paste that strongly penetrates the fabric. The greatest difference can be seen in the case of pure PEDOT:PSS paste (100:0), which has a low viscosity and the greatest penetration into the fabric. This can affect the film structure and its electrical conductivity mechanism.

Regardless of the substrate used, a decrease in TCR is observed as the bismuth telluride content of the paste increases. As can be observed, it is not linear and there are slight fluctuations in the value. This is most likely due to the inhomogeneity of the structure of the composite material, which consists of a PEDOT:PSS pop-up matrix and semiconducting grains of bismuth telluride filler. In addition, the measurement of structures made on textiles on the TCR measurement bench is problematic.

It is well known that the temperature coefficient of resistance (TCR) of conducting polymers, including PEDOT:PSS, typically exhibits a negative value, as increasing temperature significantly enhances charge carrier mobility, thereby increasing the material’s conductivity [40]. The PEDOT:PSS layers obtained from the screen-printing paste used in this study exhibit a TCR of 2343 ppm/K (approximately 0.2%/K). This value lies within the range reported in the literature and is primarily attributed to the fact that the tested material is not pure PEDOT:PSS but a screen-printing paste containing 5 wt% PEDOT:PSS (as described in the Section 2). The presence of small amounts of additional components may affect the TCR value, especially since thermosetting polymers—used as curing agents in such pastes—are typically characterized by a positive temperature coefficient of resistance. Typically, PEDOT:PSS materials are expected to have TCR values between −0.91%/K and −1.14%/K [41]. However, PEDOT:PSS is commercially available in a variety of formulations, which can significantly influence the TCR value. Through modification of the composition, PEDOT:PSS films with relatively high and even positive TCR values (∼0.6%/K at room temperature) have been reported [42].

### 3.5. Seebeck Coefficient

**Figure 8:**
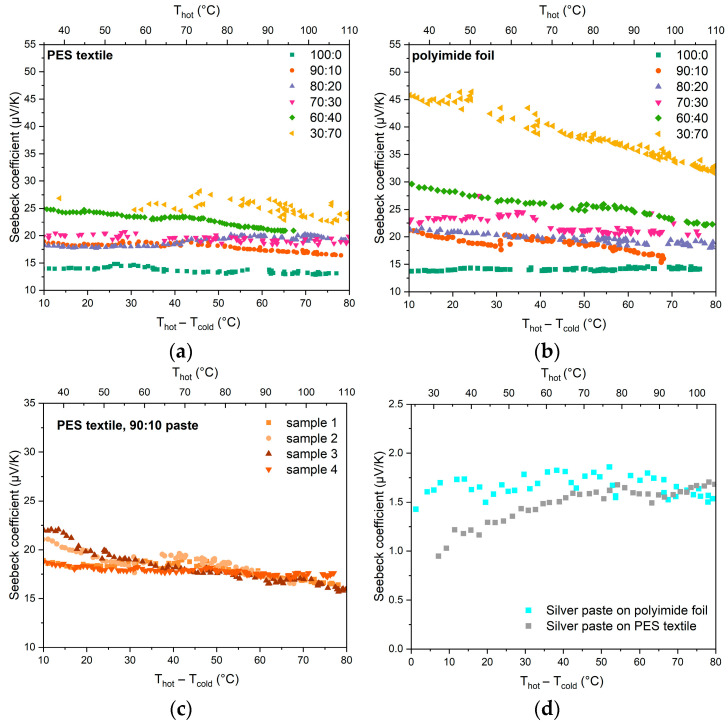
Seebeck coefficient of obtained films versus temperature gradient, for pastes printed on (**a**) PES textile and (**b**) polyimide foil. (**c**) Measurements of consecutive samples made with 90:10 paste on fabric. (**d**) Seebeck coefficient of silver paste versus temperature gradient.

The measurements showed that, irrespective of the type of substrate used the Seebeck coefficient was the smallest for the pure PEDOT:PSS paste (value of approximately 14 μV/K). This is a typical value for this material, as it is typically in the range of 15–18 µV/K [43]. The Seebeck coefficient was increased with increasing bismuth telluride content in the paste (Figure 8a,b). For Bi_2_Te_3_ the Seebeck coefficient is −170 μV/K for the n-type material and 160 μV/K for the p-type material [44]. However, as can be observed, the effect of bismuth telluride, with its much higher Seebeck coefficient, on the paste coefficient is relatively small. This is due to the lack of sufficient percolation connections by bismuth telluride molecules in the PEDOT:PSS matrix; therefore, its properties are dominant.

The PEDOT:PSS/Bi_2_Te_3_ content ratio of 30:70 wt% was the highest possible, taking into account not only the thermoelectric parameters of the obtained layers, but also their usefulness in textile printing.

However, we prepared a reference sample without PEDOT:PSS, containing 90% Bi_2_Te_3_ by weight—designated 0:90. It also contains an acrylic carrier (10% by weight) that allows for the preparation of the paste and printing on the fabric. The resulting ratio was determined experimentally: it was the smallest possible amount of carrier that would allow for obtaining a paste suitable for screen printing (although its rheology was not very good). Below is a graph of the Seebeck coefficient versus temperature difference for the prepared paste. As can be seen, the obtained value is significantly higher for the pastes made for ΔT = 60 °C:37 uV/K, significantly dependent on temperature. Its high Seebeck coefficient, especially for small temperature differences (80 uV/K for ΔT = 10 °C), is promising for energy harvesting from human–ambient temperature. However, despite such a high Seebeck coefficient, this paste had no practical applications—it was difficult to create repeatable paths from it, the layers were subject to rapid physical damage (they were brittle and fell off the fabric), oxidized (losing thermoelectric properties), and had very high resistance, resulting in a paste with a low power factor.

In all the pastes that were mixtures of the two materials, the Seebeck coefficient had a fairly stable value over the entire range of temperatures measured. In addition, the results obtained were characterized by rather good repeatability (Figure 8c). Bi_2_Te_3_ can oxidize to a small extent even at temperatures below 120 °C. However, according to the literature, at temperatures below 250 °C (523 K), oxidation, especially in n-type samples, occurs very slowly, and only above 300 °C (573 K) is protection against degradation necessary due to the increased rate of oxidation [45]. In the event of oxidation of the material, a change in the electrical and thermoelectric parameters of the material would be visible. However, the repeatability of the results indicates the stability of the electrical parameters of the paste, which can be concluded that bismuth telluride does not oxidize

Furthermore, in our work, the thermocouple arms containing bismuth telluride consist of a PEDOT:PSS-based polymer paste with bismuth telluride powder dispersed in it. As a result, even in an oxygen atmosphere, the bismuth grains have limited contact with oxygen, as the polymer carrier phase of the composite forms a protective layer for the bismuth telluride. Both during the drying of the samples and their testing, the telluride was already dispersed in the polymer matrix. A Seebeck coefficient of the silver paste (PE 874) was +1.0–+1.8 μV/K depending on temperature and substrate used (Figure 8d).

### 3.6. Power Factor

**Figure 9:**
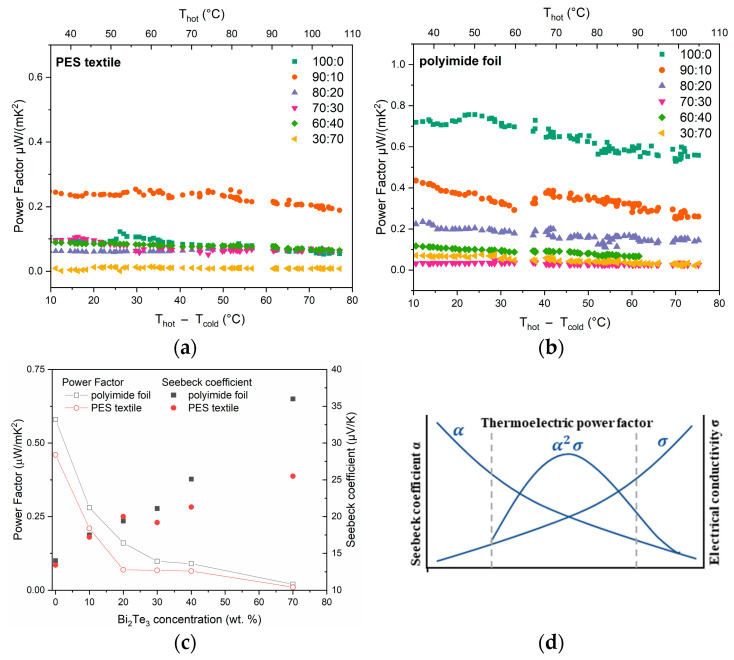
Power factor of fabricated films versus temperature gradient, for pastes printed on (**a**) PES textile and (**b**) polyimide foil. (**c**) Seebeck coefficient and power factor (at ΔT = 60 °C) as a function of paste composition. (**d**) Diagram of the dependence of thermoelectric parameters.

Based on the resistance measurements of the samples and the Seebeck coefficients, the power factor of all films and substrates was calculated (Figure 9). The suitability of materials for thermoelectric components, especially voltage generators, is assessed on the basis of the thermoelectric power factor PF [W∙m^−1^∙K^−2^] (3). As can be seen in this case, better results were also obtained for materials printed on polyimide foil. Furthermore, it is clearly visible that the addition of bismuth telluride does not positively affect the power factor. In the case of the resulting pastes, the key parameter influencing their thermoelectric utility is their electrical conductivity.

It implies that materials should have both a **high electrical conductivity** (σ) [S·m^−1^] and a **high Seebeck coefficient (α)**. These values are dependent on each other, as both depend on the amount and mobility of the carriers. For this reason, an increase in one always results in a decrease in the other (Figure 9c,d).

To produce temperature sensors, it is only important to obtain the highest possible Seebeck coefficient, while for voltage generation, it is equally important to obtain a material with the highest possible electrical conductivity. Obtaining the optimal thermoelectric material for this purpose typically involves finding compromise values that allow for the maximum power factor, which then translates into the output voltage and output power of the fabricated thermopiles.

This is of significant importance in classical thermoelectric materials and in wearable electronics, where thermopiles are considered potential power sources for wearable electronic systems. In this case, it is important to note that, in addition to optimizing thermoelectric parameters, maintaining material parameters that allow for their use in contact with fabric is also crucial.

### 3.7. Thermopiles

Thermopiles were screen-printed on textile substrates or polyimide foil. They consisted of five thermocouples, connected as in Figure 2. One arm (silver colour in Figure 2) was made from a silver-based PE 874 paste. The other was made from a paste based on the tested material, marked 100:0 (pure PEDOT:PSS) or 70:30 (PEDOT:PSS/Bi_2_Te_3_). This means that four types of thermopiles were made. The thermopiles were then characterized on the set-up shown in Figure 3b, in the temperature range from 22 °C to 125 °C. The resistance of structures made from paste based on pure PEDOT:PSS (100:0) was noticeably lower than structures made from 70:30 material. A lower resistance was also measured for structures made on polyimide foil. In the case of textiles, the paste penetrates the structure of the substrate, filling the space between the fibres throughout its thickness. As a result, it is more difficult for electrical charges to flow through the film. This can be seen in the electrical parameter shown in Table 2, where films made of the same materials had a higher resistance on textiles than on the polyimide foil.

**Figure 10:**
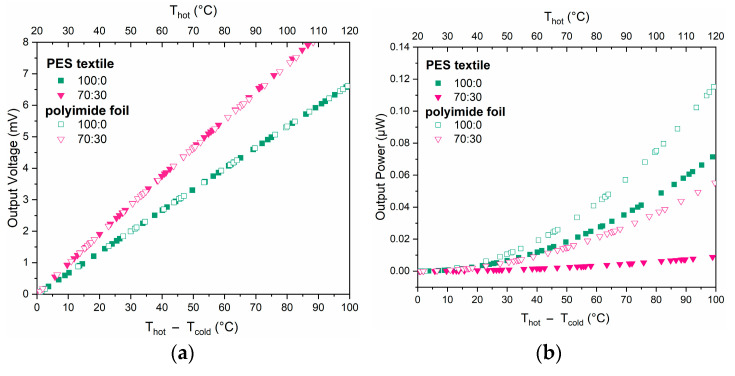
Measurements of thermopiles printed on foil and fabric substrates: (**a**) output voltage (E_T_), (**b**) output power (P_OUT_).

The output voltages of the thermopiles were 8 mV for 70:30 and 6 mV for pure PEDOT:PSS, at the temperature difference of ΔT of 100 °C (Figure 9a). This is a good starting point for further study, given that the thermopiles consisted of only five thermocouples. The higher output voltage of the Ag/70:30 structures is due to the somewhat higher Seebeck coefficient of this material relative to PEDOT:PSS (Figure 8). No significant difference was observed between the thermopiles on the textile substrate and on the polyimide foil. Higher power output was achieved by structures based on pure PEDOT:PSS due to the significantly lower resistance (Figure 10b). In this case, a clear difference can be seen between structures on polyimide and on textile.

### 3.8. Harvesting Low-Grade Thermal Energy from Human–Environment Temperature Difference

All fabricated thermopiles were tested to evaluate their applicability as voltage generators utilizing the temperature gradient between the human body and the ambient environment. The measurements were conducted on structures placed on a laboratory table, where the inner side of the hand was cyclically applied to the “hot” side of the thermopile. The graph of thermocouple measurements at the measuring station (Figure 10) shows that a higher output voltage is obtained for the 70:30 paste, but the difference between the samples is significant for higher ΔT values. In the case of low-grade thermal energy from human–environment temperature difference ΔT was less than 10 °C and for both samples and both substrates it was approximately 0.3 mV (Table 4), which is consistent with the previous results (Figure 10a). As can be seen, the output power values for samples made from PEDOT:PSS (100:0) are significantly higher regardless of the type of substrate. In addition, higher values were obtained for the same pastes, but applied to substrates made of polyimide foil, which is consistent with Figure 10b.

Table 4 summarizes the results of the maximum values obtained for the measurements of four samples, while Figure 10 presents the measurement for a selected sample so as not to obscure the results and to be able to overlay the measurement photo on it.

**Figure 11:**
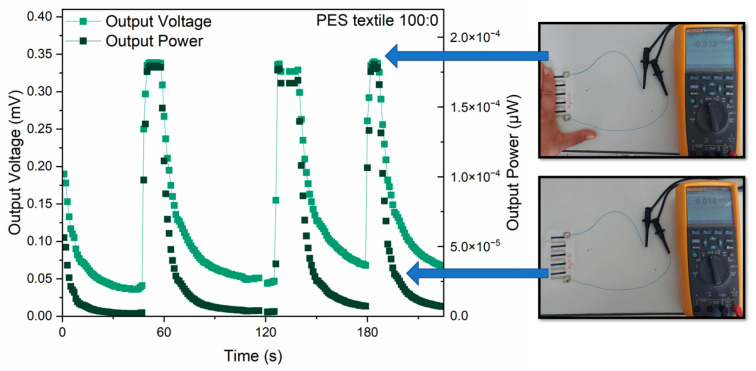
Example measurement of thermopiles printed on fabric substrates (sample 100:0) and measured in contact with human body heat with added photos of the measurement system.

Notably, during repeated measurements, the device operated reversibly and exhibited no signs of degradation, indicating good operational stability and durability (Figure 11).

## 4. Conclusions

In summary, this work has demonstrated that it is possible to fabricate thermocouples and thermopiles on flexible substrates made of film and fabric, using a thick-film technology. The thermoelectric composite materials obtained in this work were characterized by sheet resistance of 26–264 Ω/sq, Seebeck coefficient of +14–+45 μV/K and power factor of 0.05–0.8 μW/mK^2^.

The resistance/resistivity of the samples changes with the composition of the material. The higher the content of the less conductive bismuth telluride in the paste, the lower the electrical conductivity of the overall material. Furthermore, in all cases the resistivities of the pastes printed on foil and fabric are very similar, indicating that good quality prints can be obtained on textile substrates. In addition, the conductivity of the films made on the foil is somewhat higher. This is due to the better quality of the print made on the foil than on the fabric, because in the case of prints on fabric, part of the paste penetrates the fabric. The effect of the substrate is most evident in the 100:0 paste containing only pure PEDOT:PSS polymer paste. This is due to the fact that this paste has the lowest viscosity of all (no solid particles), so it penetrates the fabric volume the most (the film thickness of pure PEDOT:PSS is also by far the smallest—approximately 10 μm, while the other films are 20–70 μm).

The Seebeck coefficient of the 100:0 PEDOT:PSS paste is the most stable over time, and reproducible from one series of measurements to the next, but also the lowest of all samples at around 14 μV/K, which is in agreement with literature data [43,46]. In the case of mixing the two substances, a 30% weight addition of bismuth telluride to PEDOT:PSS already increases the Seebeck coefficient significantly, which increases linearly with increasing Bi_2_Te_3_ content up to a value of about 45 μV/K. This increase is relatively small considering that the Seebeck coefficient of volumetric p-type bismuth telluride is about 200 μV/K at room temperature [47]. However, it must be taken into account that in the fabricated paste enabling printing on fabric, bismuth telluride is added as powder with a grain size of 5–50 μm, making the dominant influence on the Seebeck coefficient the presence of PEDOT:PSS, which forms the matrix containing the bismuth telluride powder. However, percolation paths are possible in most of the pastes made, while the tunnelling conductive layers of the polymer are always a significant contributor.

An inverse relationship is observed for changes in material resistance. The PEDOT:PSS polymer paste has the lowest sheet resistance of 26 Ω/sq, while the addition of increasing amounts of the semiconducting bismuth telluride causes the resistance to increase increasingly up to a value of 264 Ω/sq.

The value that determines the suitability of the materials for making thermoelectric components from them is the power factor combining the two. In the case of the samples obtained, its value decreases significantly with the bismuth telluride content of the paste. This indicates that the predominant significance in thermoelectric applications of the fabricated pastes has the resistivity and not their Seebeck coefficient. For this reason, further work is planned to design materials that can be used in wearable electronics, but with a higher PF value. Such results can be achieved, among others, by doping Bi_2_Te_3_ with antimony (Sb), which allows a Seebeck coefficient of 220 µV/K to be achieved at room temperature [48], or by using more finely divided bismuth telluride particles, which will allow the telluride particles distributed in the polymer matrix to exceed the percolation threshold more quickly. This may increase the Seebeck coefficient of the paste with a lower bismuth telluride content in PEDOT:PSS, and without a significant increase in resistance. It can also be important to doping pastes with carbon nanoparticles, which significantly alter the Seebeck coefficient of materials: doping with graphene (α = −150 µV/K) [49], carbon nanotubes (α = −130 µV/K) [50], single wall carbon nanotubes (α = −140 µV/K) [51].

Two types of thermopiles consisting of five thermocouples were fabricated and characterized. In both, one arm was made from Ag-based paste, and the other from pure PEDOT:PSS (characterized by the highest PF ratio) or from 70:30-based paste. The determined Seebeck coefficient level for Ag paste was +1.5–+1.8 μV/K for PEDOT:PSS, +14 μV/K and for 70:30 +20–+21 μV/K (Figure 7). This means that the expected Seebeck coefficient of whole thermopiles was about 62 μV/K for PEDOT:PSS/Ag and about 95 μV/K for 70:30/Ag, which coincides with the measured results (Figure 9). The thermopiles were fabricated on both substrates’ polyimide foil and fabric. No significant difference in the level of generated output voltage was observed between the thermopiles on the different substrates. This was expected, as it was due to the peculiarities of the Seebeck phenomenon, which does not depend on the geometry or dimensions of the structures. The best output performance was measured for PEDOT:PSS/Ag thermopile on the polyimide foil, which generated a voltage of 8 mV and a power of 0.10 μW at a temperature gradient of 100 °C along the structure. For the textile substrate, the same output voltage was achieved, but lower power (0.07 μW) due to higher internal resistance. Its reduction will be one of the key objectives of further research. The developed generators demonstrated promising results, with a generated voltage of approximately 0.3 mV and output power ranging from 3.7 × 10^−6^ to 2.8 × 10^−4^ μW, depending on the structure. This performance makes them well-suited for wearable electronics, enabling the powering of low-energy sensors, communication modules, or physiological monitoring systems. Their stable operation and compact form factor further support seamless integration into clothing or wearable accessories.

## Figures and Tables

**Table 1:** Composition of the received pastes.

Weight Ratio of PEDOT:PSS/Bi_2_Te_3_ Sample Description	Concentration PEDOT:PSS wt.%	Concentration Bi_2_Te_3_ wt.%
100:0	100	0
90:10	90	10
80:20	80	20
70:30	70	30
60:40	60	40
30:70	30	70

**Table 2:** Comparison of resistance per sheet R□ and resistivity.

Sample Description	Sheet Resistance [Ω/sq]		Resistivity [Ω·m]		Film Thickness [mm]	
	PES Textile	Polyimide Foil	PES Textile	Polyimide Foil	PES Textile	Polyimide Foil
100:0	26	33	0.0013	0.00039	0.051	0.012
90:10	23	38	0.0014	0.0017	0.062	0.043
80:20	62	47	0.0034	0.0018	0.056	0.037
70:30	63	65	0.0045	0.0022	0.071	0.034
60:40	152	152	0.014	0.0091	0.090	0.059
30:70	232	333	0.027	0.026	0.116	0.078
PE 874	0.22	0.12	0.00001	0.000005	0.045	0.040

**Table 3:** Temperature coefficient of resistance of obtained materials.

Sample Description	TCR (ppm/K)	
	PES Textile	Polyimide Foil
100:0	2343	1267
90:10	107	84
80:20	127	144
70:30	255	200
60:40	−145	−92
30:70	−183	−413

**Table 4:** Energy harvesting from human–ambient temperature gradient.

Sample Description		Output Voltage [mV]	Output Power [μW]
PES textile	100:0	0.34	1.8 × 10^−4^
	70:30	0.34	3.7 × 10^−6^
polyimide foil	100:0	0.33	2.8 × 10^−4^
	70:30	0.31	23.9 × 10^−6^

## Data Availability

The original contributions presented in this study are included in the article. Further inquiries can be directed to the corresponding author.
